# Genetic Analyses on Transgender Individuals: Impact on Physician Attitudes and Surgical Decision‐Making

**DOI:** 10.1002/brb3.70228

**Published:** 2025-01-09

**Authors:** Duygu Onur Cura, Ayfer Ülgenalp, Tufan Çankaya

**Affiliations:** ^1^ Department of Molecular Medicine, Institute of Health Sciences Dokuz Eylül University Izmir Türkiye; ^2^ Department of Medical Genetics, Faculty of Medicine Dokuz Eylül University Izmir Türkiye

**Keywords:** attitudes of physicians, gender confirmation surgery, genetic analyses, transgender and gender diverse

## Abstract

**Purpose:**

Genetic studies on the transgender and gender diverse (TGD) community have started to appear in the literature. However, there are limited studies on how genetic data will impact attitudes and perspectives toward TGD individuals. In this study, we investigated the impact of genetic alterations on physicians' attitudes toward TGD individuals and on physicians' decisions concerning gender confirmation surgery (GCS). In this context, we intended to highlight a number of strategies to reduce the inequalities that the TGD community is exposed to in accessing health‐care services.

**Method:**

An online survey including the Turkish version of the Attitudes Toward Transgendered Individuals Scale (ATTIS) was completed by 224 physicians from relevant specialties. Scheffé and least significant difference (LSD) post hoc analysis methods were used to determine physicians' perspectives on whether genetic findings would cause TGD individuals to feel validated/invalidated. Multivariate multinomial logistic regression analysis was employed to assess their responses concerning the decision to perform GCS when genetic alterations had been identified.

**Results:**

More than half of the physicians expressed the view that genetic analyses for TGD individuals would confer benefits (67.1%). Those who thought that the presence of gender–diversity‐related genetic alterations would have a “positive impact” on their GCS‐related decision to operate were found to have less positive attitudes toward TGD individuals (Bonferroni corrected *p* < 0.001). Multivariate multinomial logistic regression analysis revealed that age, presenting the odds ratio as the strongest factor, distinguished the “no impact” group from the reference “positive impact” group, particularly among those aged ≤ 35 years (3.299, 95% CI: 1.355–8.033; *p* = 0.009).

**Conclusion:**

Although genetic analysis of TGD individuals is predicted to have a positive effect on physicians' attitudes toward them and on the GCS decision‐making process, it should be emphasized that the benefits for TGD individuals must outweigh the potential harm. The results showed that physicians need “early and continuing education” to develop a comprehensive perspective on gender identity. The most appropriate approach for genetic testing would be to include the TGD community in decision‐making processes and to develop guidelines for the interpretation of genetic data.

## Graphical abstract

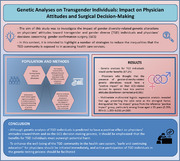

## 1 Introduction

The term “transgender and gender diverse (TGD)” refers to individuals whose gender identity differs from the social and cultural expectations associated with the sex they were assigned at birth (Coleman et al. 2022). While biological sexual development is primarily determined by genetic and hormonal factors, gender identity is a complex and multifaceted construct shaped by environmental and cultural factors, in addition to its biological basis (Rosenthal 2021).

Genetic explanations for TGD identities are variable and complex, with few studies in the literature on this subject. Chromosomal analyses have found higher rates of Klinefelter syndrome among TGD individuals relative to the general population, although only a limited number of TGD individuals with Klinefelter syndrome have been documented (Cankaya et al. 2021). Genetic alterations have also been detected in microarray (Fernández, Guillamon, Gomez‐Gil, et al. 2018) and whole‐exome/genome sequence (Yang et al. 2017) analyses, but it remains very difficult to confirm whether the detected genetic alterations are directly related to TGD. In another whole‐exome sequence analysis, it was determined that some variants obtained were related to estrogen receptor‐activated pathways of sexually dimorphic brain development (Theisen et al. 2019). Other studies have investigated variants affecting the expression of genes associated with sex hormones (Lombardo et al. 2013; Ujike et al. 2009; Fernández Guillamon, Cortés‐Cortés, et al. 2018; Foreman et al. 2019). It would be appropriate to conduct comprehensive genetic research on TGD individuals by taking advantage of advanced technological developments, while being aware that genetic alterations may only form the infrastructure of this complex trait.

To be considered for gender confirmation surgery (GCS) in Turkey, TGD individuals are required to be evaluated by a medical board comprising physicians from pertinent specialties, particularly psychiatry, and plastic surgery. The decisions made by the board physicians wield considerable influence in facilitating both the surgical and legal processes. The thoughts and attitudes of physicians toward TGD individuals influence the physicians' decisions regarding GCS. Therefore, as data on the genetic basis of gender diversity have the potential to influence physicians' GCS decision‐making process, this study assessed physicians' perspectives on genetic studies on TGD individuals, the potential impact of genetic alterations on physicians' decisions regarding GCS, and the relationship between such variants and physicians' attitudes. In doing so, we aim to contribute to strategies that improve health‐care services for the TGD community by emphasizing the potential benefits and harm that data from genetic research may offer to these individuals through physicians' attitudes.

## 2 Methods

### 2.1 Data and Sampling

This study was based on a survey administered to physicians between July 22, 2022 and September 22, 2022. An online survey methodology was used and Google Forms served as the survey platform. The link to the questionnaire was delivered via different channels including WhatsApp, Telegram, and email. The recruitment of participants was well targeted by sharing the survey link through relevant professional networks and groups before the questionnaire was distributed. Turkish physicians' participated on a voluntary basis and included specialists in psychiatry, gynecology, medical genetics, urology, plastic surgery, and endocrinology who potentially collaborate with TGD individuals during the GCS process. Specialty groups were combined to obtain statistical significance. Psychiatry, medical genetics, and endocrinology specialties were gathered under the heading of “internal branch,” and gynecology, urology, and plastic surgery under “surgical branch.”

The first section of the questionnaire consisted of 11 questions evaluating the sociodemographic data of the participants (age, sex, specialty, seniority, duration of practice), their thoughts on genetic analysis of TGD individuals, and the impact of those thoughts on the decision to perform GCS in cases of identified gender–diversity‐related genetic alterations. In the second section of the questionnaire, the Turkish version of the Attitudes Toward Transgendered Individuals Scale (ATTIS) (Gölge and Akdemir 2019) was administered to the participants to evaluate their current attitudes toward TGD individuals and the effect of the attitudes on the decision to operate. The study was approved by the Ethics Committee of Dokuz Eylul University (approval number: 2022/23‐07).

### 2.2 Outcome Variables

The outcome variables for this study focused on assessing how genetic factors affected physicians' attitudes toward TGD individuals and the physicians' decision‐making processes for GCS.

*ATTIS*: In addition to determining the attitudes of physicians, the Turkish version of ATTIS (Gölge and Akdemir 2019) was used to evaluate the relationship between their attitudes and the effect of genetic testing on surgical decision‐making. This scale was selected as it is easy to apply and understand, and has proven validity and reliability. The ATTIS consists of 20 items scored on a 5‐point Likert scale, higher scores indicating more positive attitudes.

*The impact of genetic data on surgical decision‐making*: Physicians indicated whether a genetic explanation for TGD individuals would impact their GCS‐related decision to operate with response options of “positive,” “negative,” “no impact,” and “not sure.”

### 2.3 Covariates

The covariates consisted of the variables of age (≤ 35 years old/> 35 years old), sex (male/female), specialty (internists/surgeons), duration of practice (≤ 15 years/> 15 years), views on the performance of genetic analyses on TGD individuals (beneficial/harmful/meaningless/no idea), and knowledge of the genetic basis of gender diversity (environmental/both genetic and environmental).

### 2.4 Statistical Analysis

Using the open‐source software OpenEpi v.3, the minimum sample size required to provide 80% power with an effect size of 0.5 and a confidence limit of 0.05 was calculated to be 162 subjects. Statistical analyses were performed using IBM SPSS v.24 software (SPSS Inc., Chicago, IL, USA). Descriptive statistics were presented as number (*n*) and percentage (%). The *χ* ^2^ test or Fisher exact test was used to measure categorical variables. Comparisons of measurement values in more than two groups were made using a *t*‐test. The Kruskal–Wallis test and the Mann–Whitney *U* test were applied in the assessments of physicians' attitudes in the presence of gender–diversity‐related genetic alterations, in respect of both surgical decision‐making and the physicians' views on whether TGD individuals would feel validated or invalidated by the detection of genetic alterations.

In evaluating physicians' attitudes toward TGD individuals feeling validated/invalidated in the presence of gender–diversity‐related genetic alterations, values of *p* = 0.05/3 = 0.0167 were determined after Bonferroni correction, and in the evaluation of responses regarding the GCS‐related decision to operate when gender–diversity‐related genetic alterations were identified, values of *p* = 0.05/6 = 0.008 were determined after Bonferroni correction. The attitudes of physicians who thought TGD individuals would feel validated/invalidated in the presence of gender–diversity‐related genetic alterations were calculated using one‐way analysis of variance (ANOVA). To determine which groups caused the difference, the Scheffé and the least significant difference (LSD) methods were applied from post hoc analyses. Multivariate multinomial logistic regression analysis was used to evaluate the responses of the participants related to the GCS‐related decision to operate in the presence of genetic alterations. A value of *p* < 0.05 was considered statistically significant.

## 3 Results

### 3.1 Characteristics of Participants

The survey completion rate was 97.8%, with 219 of the 224 participants providing complete responses (123 females, 96 males, and 0 others). The majority of the participants (90.4%, *n* = 198) were aged ≤ 45 years, 136 (62.1%) were internal medicine specialists, 83 (37.9%) were surgical specialists, and 40 (18.3%) had > 15 years of practice. All characteristics of the study participants are presented in Table 1.

**TABLE 1:** Characteristics of the participants.

	*n*	%
Sex (female/male/other)	123/96/0	56.2/43.8/0
Age 25–35 36–45 > 45	108 90 21	49.3 41.1 9.6
Specialty Endocrinology Gynecology Medical genetics Plastic surgery Psychiatry Urology	3 53 67 14 66 16	1.4 24.2 30.6 6.4 30.1 7.3
Seniority Residents Specialist Lecturers	34 138 47	15.5 63 21.5
Duration of practice < 5 years 5–15 years 16–25 years > 25 years	49 130 30 10	22.4 59.4 13.7 4.6

### 3.2 The Turkish Version of the ATTIS

**FIGURE 1:**
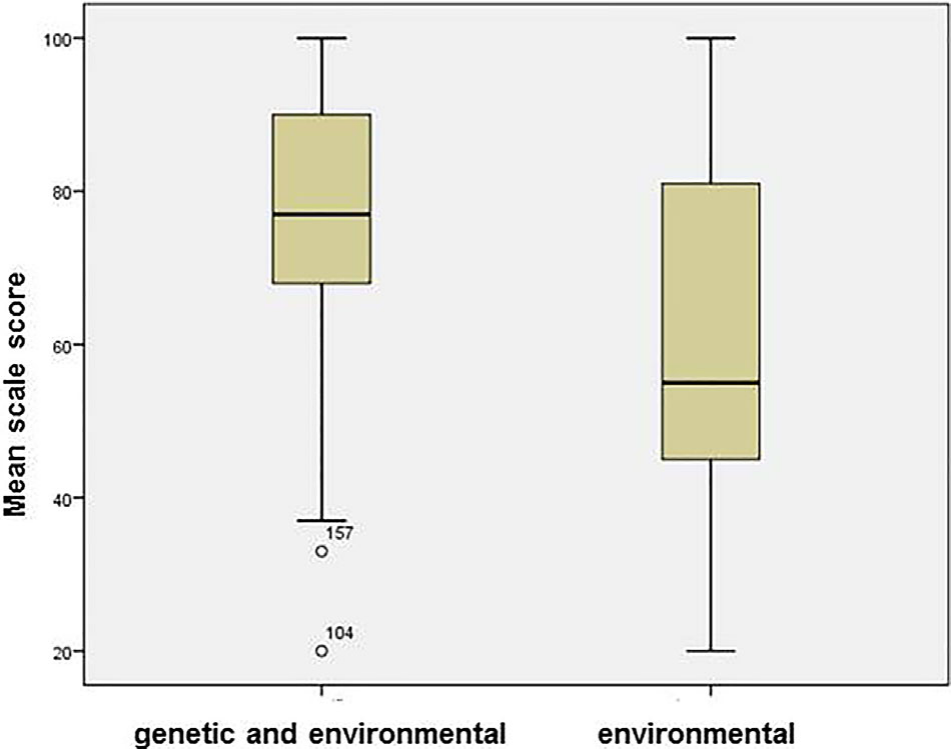
Box plots displaying the mean ATTIS scores based on physicians' view about the cause of gender diversity.

The mean scale score was found to be 74.39 ± 17.632 (*n* = 219). When the scale data of the physicians were compared, no significant difference was observed in terms of age, sex, academic title, duration of practice, and perspective on the performance of genetic analyses on TGD individuals (*p* > 0.05). Physicians who thought that TGD identities arise from genetic and environmental factors (M = 76.11 ± 1.146, *n* = 198) had a more positive attitude toward TGD individuals than physicians who thought that it arose only from environmental factors (M = 58.24 ± 4.997, *n* = 21) (*p* = 0.001, *r* = 0.23) (Figure 1). A statistically significant difference was found between the mean scale scores based on physicians' opinions on whether genetic research would make TGD individuals feel validated, with a medium to large effect size [*F*(2, 216) = 8.82, *p* < 0.001, *η* ^2^ = 0.075]. The physicians who thought that TGD individuals would feel validated by the detection of genetic alterations were found to have less positive attitudes toward TGD individuals (M = 70.39, SD = 16.936) compared to those who did not agree with this idea (M = 81.40, SD = 17.591) (both Scheffé and LSD: *p* < 0.001). A statistically significant difference was found between the mean scale scores of the latter groups despite the moderate effect size of the physicians who thought that conducting genetic research may result in TGD individuals feeling invalidated [*F*(2, 216) = 5.701, *p* = 0.004, *η* ^2^ = 0.050]. Post hoc comparisons showed that physicians who thought that TGD individuals would feel invalidated due to the detection of genetic alterations had more positive attitudes toward TGD individuals (M = 80.66, SD = 18.428) than physicians with the opposite view did (M = 71.49, SD = 17.090) (Scheffé: *p* = 0.004, LSD: *p* = 0.001).

### 3.3 Influence of Genetic Findings on the Decision‐Making Process for GCS

A total of 55.7% (*n* = 122) of the study participants stated that the detection of gender–diversity‐related genetic alterations would exert a “positive impact” on the decision to operate for GCS [negative impact 2.3% (*n* = 5), no impact 35.6% (*n* = 78), unsure 6.4% (*n* = 14)]. A *χ* ^2^ independence test was applied to examine the relationship between age group and the decision to operate in the presence of gender–diversity‐related genetic alterations. A significant association was determined (*χ* ^2^(3, *N* = 219) = 17.257, *p* = 0.001), with a small to moderate effect (Cramér's *V* = 0.162). This demonstrated that if genetic alterations were detected, the GCS‐related decision to operate of physicians aged > 35 years would be positively impacted compared to that of younger physicians. Duration of practice and the decision to operate in the presence of gender–diversity‐related genetic alterations were determined to be significantly associated (*χ* ^2^(3, N = 219) = 8.597, *p* = 0.035), although the effect size was small (Cramér's *V* = 0.198), indicating a moderate correlation. These findings were supported by Monte Carlo simulations (*p* = 0.036). Physicians with less than 15 years of professional experience reported that the presence of genetic alterations would have no impact on their GCS‐related operating decision, more frequently than more experienced physicians did. A *χ* ^2^ test of independence showed a significant association between specialty and GCS‐related decision to operate in the presence of genetic alterations, with a small effect size (*χ* ^2^(3, *N* = 219) = 10.997, *p* = 0.012, Monte Carlo *p* = 0.013, Cramér's *V* = 0.158). A significant difference was detected between surgeons and internists, who reported that any detected genetic alterations would have “no impact” on their GCS‐related decision to operate (Table 2).

**TABLE 2:** Impact of gender–diversity‐related genetic alterations on physicians' GCS‐related decision to operate by age, sex, specialty, and duration of practice.

	Positive *n* (%)	Negative *n* (%)	No impact *n* (%)	Unsure *n* (%)	*p* value
Age ≤ 35 ages > 35 ages	44 (42.3) 78 (67.8)	4 (3.8) 1 (0.9)	50 (48.1) 28 (24.3)	6 (5.8) 8 (7)	< 0.001^a^
Sex Female Male	63 (51.2) 59 (61.5)	3 (2.4) 2 (2.1)	47 (38.2) 31 (32.3)	10 (8.1) 4 (4.2)	> 0.05
Specialty Internists Surgeons	64 (47.1) 58 (69.9)	4 (2.9) 1 (1.2)	58 (42.6) 20 (24.1)	10 (7.4) 4 (4.8)	0.01^a^
Duration of practice ≤ 15 years > 15 years	95 (77.9) 27 (22.1)	4 (80) 1 (20)	71 (91) 7 (9)	9 (64.3) 5 (35.7)	0.036^b^

^a^ Between positive and not affected.

^b^ Between not affected and no idea.

### 3.4 Multivariate Multinomial Logistic Regression for the Impact of Genetic Alterations on the Participants' Decision to Operate

**FIGURE 2:**
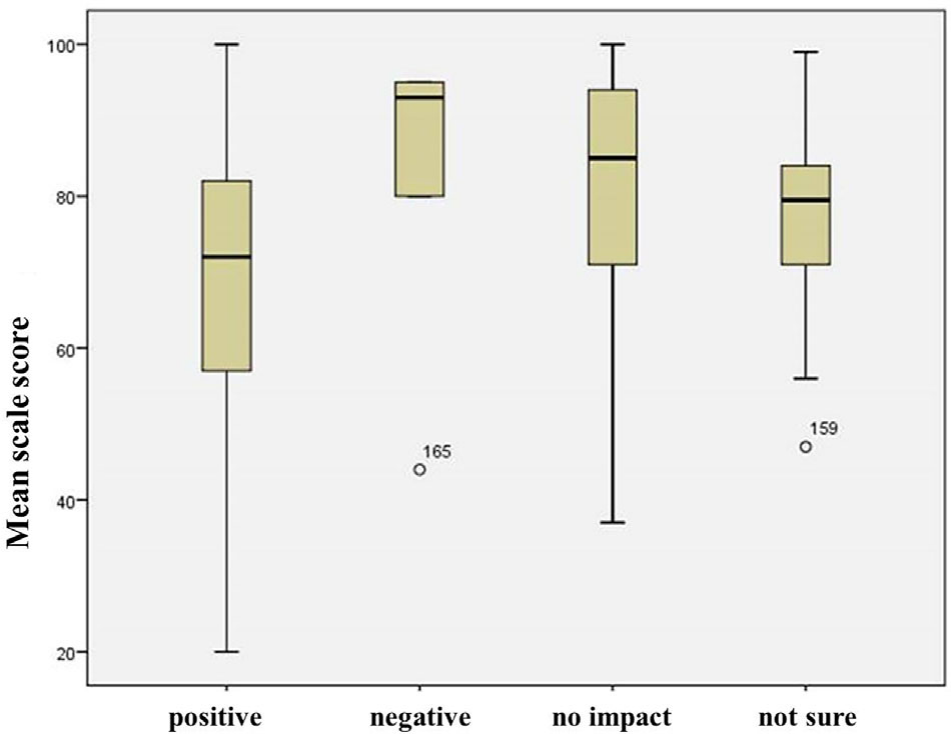
Box plots displaying the mean ATTIS scores based on physicians' GCS‐related decision to operate in the presence of gender–diversity‐related genetic alterations.

Significant findings were determined using multinomial logistic regression (*χ* ^2^(39) = 149.946, *p* < 0.001, Nagelkerke *R* ^2^ = 0.582), with the model successfully predicting 74.9% of cases. The study participants who did not think there would be a “positive impact” on their surgical decision‐making were more likely not to express an opinion on genetic analysis in TGD individuals (*p* < 0.05). Younger physicians were found to be 3.29 times more likely to state that the detection of genetic alterations would not affect their GCS‐related decision to operate (95% CI: 1.355–8.033, *p* = 0.009) (Table 3). Based on the results of the Mann–Whitney *U* test analysis, the attitude scale mean value of those who stated that gender–diversity‐related genetic alterations would have a “positive impact” on their GCS‐related decision to operate (Mdn = 72, *n* = 122) was found to be lower than that of those who stated that there would be “no impact” on their decision (Mdn = 78, *n* = 85) (*U* = 427.500, *Z* = −1.289, Bonferroni corrected *p* < 0.001), with a small‐to‐moderate effect size (Cohen's *d* = 0.359) (Figure 2).

**TABLE 3:** Multivariate multinomial logistic regression analysis for physicians' GCS‐related decision to operate in the presence of gender–diversity‐related genetic alterations.

Variables	Negative (*n* = 5; 2.3%)			No impact (*n* = 78; 35.6%)			Unsure (*n* = 14; 6.4%)		
	B	OR (95% CI)	*p* value	B	OR (95% CI)	*p* value	B	OR (95% CI)	*p* value
≤ 35 ages (reference ≥ 35 ages)	18.112	—	>0.05	1.194	3.299 (1.355–8.033)	0.009	0.537	1.710 (0.305–9.589)	0.542
Sex (reference = female)	0.259	1.296 (0.149–11.256)	0.814	−0.212	0.809 (0.359–1.824)	0.610	−0.833	0.435 (0.100–1.884)	0.266
Specialty (reference = surgeons)	0.770	2.160 (0.186–25.120)	0.538	0.470	1.600 (0.695–3.681)	0.269	−0.105	0.900 (0.179–4.522)	0.898
Duration of practice (reference ≥ 15 years)	18.037	—	>0.05	0.527	1.693 (0.441–6.497)	0.443	−1.654	0.191 (0.029–1.276)	0.088
ATTIS score	0.056	1.058 (0.989– 1.130)	0.10	0.049	1.05 (1.021–1.080)	0.001	0.029	1.029 (0.976–1.086)	0.286
Physicians' view about the cause of gender diversity (reference = environmental)	−2.171	0.114 (0.03–4.049)	0.233	0.264	1.302 (0.283–5.982)	0.735	2.229	—	0.205
Genetic analyses are beneficial (reference = no idea)	−2.885	0.56 (0.003–1.133)	0.06	−2.021	0.133 (0.031–0.563)	0.006	−3.381	0.034 (0.005–0.225)	<0.001
TG people will feel validated (reference = not agree) Agree No idea	−1.237	0.290 (0.26–3.221)	0.314	−1.460 −0.276	0.232 (0.086–0.626) 0.759 (0.189–3.054)	0.004 0.698	−0.251 1.389	0.778 (0.078–7.718) –	0.830 0.252
TG people will feel invalidated (reference = not agree) Agree No idea	−0.34 −17.909	0.967 (0.082–11.370) –	>0.05 >0.05	−0.271 0.711	0.763 (0.260–2.237) 2.036 (0.585–7.080)	0.622 0.264	0.314 1.943	1.369 (0.190–9.877) –	0.755 0.038

Abbreviations: ATTIS, Attitudes Toward Transgender Persons Scale; TG, transgender “positive” is the reference category.

### 3.5 Physicians' Knowledge of the Genetic Aspects of Gender Diversity and an Overview of the Impact of Genetic Data on TGD Individuals

The proportion of physicians who thought that genetic analyses would be beneficial for TGD individuals was 67.1% (*n* = 147) [pointless 24.2% (*n* = 53), no idea 8.7% (*n* = 19)], and that of physicians who thought genetic analyses would make these individuals feel validated was 55.7% (*n* = 122) [not agree 28.8% (*n* = 28), no idea 15.5% (*n* = 34)]. It was ascertained that, compared to their younger counterparts, physicians aged > 35 years were more likely to think that TGD individuals would feel validated if they had gender–diversity‐related genetic alterations (*p* = 0.045) (Table 4).

**TABLE 4:** An overview of physicians' thoughts on the feelings of TGD individuals in the presence of gender–diversity‐related genetic alterations.

	Feel validated			*p* value	Feel invalidated			*p* value
	Agree	Not agree	No idea		Agree	Not agree	No idea	
Age ≤ 35 ages > 35 ages	49 (47.1) 73 (63.47)	37 (35.6) 26 (22.6)	18 (17.3) 16 (13.9)	0.045^a^	28 (26.9) 30 (26.1)	58 (55.8) 74 (64.3)	18 (17.3) 11 (9.6)	0.207
Sex Female Male	57 (59.4) 65 (52.8)	25 (26) 38 (30.9)	14 (14.6) 20 (16.3)	0.62	31 (25.2) 27 (28.1)	76 (61.8) 56 (58.3)	16 (13) 13 (13.5)	0.864
Specialty Internists Surgeons	68 (50) 54 (65.1)	45 (33.1) 18 (21.7)	23 (16.9) 11 (13.2)	0.088	45 (33.1) 13 (15.7)	70 (51.5) 62 (74.7)	21 (15.4) 8 (9.6)	0.003^a^
Duration of practice ≤ 15 years > 15 years	97 (54.2) 25 (62.5)	54 (30.2) 9 (22.5)	28 (15.6) 6 (15)	0.582	46 (25.7) 12 (30)	108 (60.3) 24 (60)	25 (14) 4 (10)	0.734

^a^ Between agree and not agree *p* < 0.05.

**FIGURE 3:**
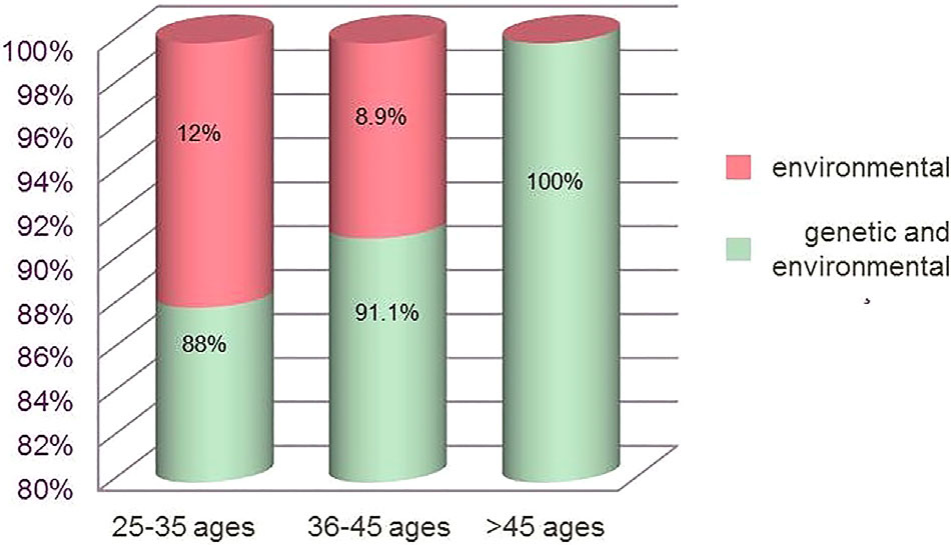
Physicians' perspectives on the etiology of gender diversity according to age groups.

Of the total respondents, 90.4% (*n* = 198) thought that both genetic and environmental factors play a role in the etiology of gender diversity, and the proportion of respondents with this view was found to increase with age (*p* > 0.05) (Figure 3). Physicians who believed that the cause of gender diversity is entirely environmental, stated that factors such as psychological trauma, upbringing, environmental impositions, and encouragement through social media play an important role.

## 4 Discussion

From a social perspective, the potential advantages and disadvantages of genetic alteration determinations for TGD individuals are a subject of ongoing debate. In a study with a broad perspective, it was shown that in the presence of a biological basis for gender identity, LGBTQ+ individuals tend to participate more in genetic research, and, simultaneously, heterosexual, and cisgender individuals, who are less tolerant of sexual and gender minorities (SGM), display more positive views toward SGM (Thomas et al. 2020). In this study, more than half of the physicians reported that gender–diversity‐related genetic alterations would positively affect their GCS‐related decision to operate, and that genetic testing would be beneficial for TGD individuals and cause the latter to feel validated. Although genetic data have the potential to have positive effects for the TGD community, genetic analyses carry the risk of reinforcing discrimination and stigmatization against these individuals due to the possibility of misuse of genetic data.

Nevertheless, a qualitative analysis evaluating the individual's own perspectives on the genetic analyses to be made emphasized that the potential genetic insights may lack sufficient utility to offset the attendant costs of acquiring this knowledge (Rajkovic et al. 2022). In another study conducted by Theisen et al. (2024), in which the thoughts and opinions of 409 TGD individuals regarding genetic testing were evaluated, the majority of individuals thought that genetic research would have positive effects on the acceptance of TGD identities and on the improvement of medical care provided by physicians, while a significant proportion thought that it could cause a risk of stigmatization and exclusion. Although we concluded that the majority of physicians thought genetic analysis could have positive effects for TGD individuals in the current study, previous studies emphasize the need to include TGD individuals in the decision to implement genetic analyses that have the potential to access data regarding the autonomy and privacy of individuals. It has even been reported that it would be beneficial to have TGD individuals as researchers in biomedical studies (Cargill 2020).

In the current study, a small number of physicians attributed the cause of gender diversity solely to environmental factors and these physicians held less positive attitudes toward TGD individuals. Although physicians know that gender development is shaped by both biological factors and environmental factors, the fact that some attribute it only to environmental factors suggests they are influenced by individuals lacking this knowledge. This collective perspective may exemplify the theory of “social contagion.” Updating information through training on the development of gender identity could contribute to breaking this erroneous perspective.

The current study's findings showed a pattern of physicians over the age of 35 being more likely to report that genetic data would have a “positive impact” on their surgical decision‐making. These physicians also appeared to be more likely to report that TGD individuals would feel validated by the detection of gender–diversity‐related genetic alterations. In contrast, physicians with less than 15 years of professional experience were found to be more likely to report that genetic data would not have an impact on their decision to operate. In a conservative society, it is not unexpected for older physicians to seek a genetic basis for their surgical decisions. However, physicians also have the potential to use such genetic profiles to pathologize TGD individuals. Therefore, there is a need to emphasize the importance of education to direct the attitudes and perspectives of society, especially physicians, in a positive direction (Cutillas‐Fernandez et al. 2023). A previous study highlighted that the effectiveness of cultural competence training against transphobia is insufficient (Stroumsa et al. 2019). In order to prevent discrimination against TGD individuals, it is recommended to start “early medical education” during medical school and continue the process with “continuing medical education” (Vijay et al. 2018). Moreover, in accordance with the contact hypothesis (Allport 1954), implementing practices that allow physicians to interact with TGD individuals can promote the reduction of prejudice and ameliorate mutual relationships. This would prompt improved attitudes toward TGD individuals.

The fact that genetic explanations affected GCS‐related decision to operate more in surgeons than in internal medicine physicians also supports this hypothesis and emphasizes the need for “continuing medical education.” This approach may be related to surgeons viewing genetic information as a biological justification. However, this may create the untrue impression that TGD individuals need a genetic basis on which to verify their identity. Emphasizing the fact that genetic factors are only one of the effects on an individual's gender identity within the scope of “continuing medical education” may contribute to physicians' more balanced and individual‐focused evaluations of genetic data. This approach may facilitate access to necessary medical care for TGD individuals, ensuring it is provided without discrimination.

In this study, we observed that participants with less positive attitudes toward TGD individuals anticipated that genetic studies would make these individuals feel validated and therefore presumed that there would be a “positive impact” of the detection of genetic alterations on the decision‐making process for GCS. These physicians with less positive attitudes may be willing to have genetic testing performed because they regard this condition as pathological, with the expectation that genetic data will support this view, but ignoring the complex nature of gender development. It is clear that this reductionist approach, which focuses solely on genetic factors, may negatively impact TGD individuals. Alternatively, these physicians may be searching for a biological basis on which to foster a positive attitude toward TGD individuals. In contrast, participants with more positive attitudes stated that genetic analyses may induce feelings of invalidation in TGD individuals and that the participants' GCS‐related decision to operate would remain unaffected even if gender–diversity‐related genetic alterations were detected. It can be inferred that these participants are predisposed to making affirmative decisions regarding surgery, and therefore anticipate that the genetic data will not sway their decisions. Furthermore, it can be inferred that these physicians are concerned that genetic data may induce discrimination and stigmatization against TGD individuals. Genetic data have the potential to foster a positive outlook among individuals with fixed beliefs when considering TGD individuals. However, there is also the risk of interpreting potential variants as pathogenic alterations. Interpretation of these variants requires nuance. It is clear that genetic guidelines used for diseases are not suitable for interpreting TGD individual data, and specific guidelines are needed for such cases (Theisen and Amarillo 2021).

The most significant limitation of this study was that all specialization groups could not be reached at a sufficient level. There is a need for further studies with broader participation, particularly engaging more homogeneous expert groups, to be able to obtain more robust outcomes. While this research incorporated the Turkish version of the ATTIS scale, other questions had not been previously tested for validity. This is because no previous study in the literature has investigated the effect of genetic analyses on physicians' surgical decisions regarding TGD individuals. As the data obtained in this study had a low‐medium effect size, more comprehensive research is needed to support the findings. The inclusion of TGD individuals in the study design, incorporating their views on genetic analysis, would have improved the utility of the study.

## 5 Conclusions

This study investigated the impact of genetic data, on physicians' attitudes toward TGD individuals and on the physicians' surgical decision‐making. Although genetic analysis is likely to generate positive effects on attitudes and surgical decisions, it is necessary to approach genetic data with caution to avoid nourishing reductive or pathological views. The findings suggest that some doctors may seek to base their decisions on genetic explanations, highlighting the need for comprehensive training in gender identity development. Moreover, in order to understand their perspectives, the active participation of TGD individuals in the design of genetic analyses is essential. Future studies should aim to develop specific guidelines for the interpretation of genetic data of TGD individuals. Planning and implementation of “early medical education” and “continuing medical education” to encourage inclusive attitudes should be a priority. This will be a further step toward access to ideal health‐care for the TGD community.

## Author Contributions

**Duygu Onur Cura**: conceptualization, data curation, formal analysis, visualization, writing–original draft, writing–review and editing. **Ayfer Ülgenalp**: conceptualization, supervision, writing–review and editing. **Tufan Çankaya**: conceptualization, resources, supervision, writing–review and editing.

## Conflicts of Interest

The authors declare no conflicts of interest.

### Peer Review

The peer review history for this article is available at https://publons.com/publon/10.1002/brb3.70228

## Data Availability

The data that support the findings of this study are available from the corresponding author upon reasonable request.
